# RACE AND SEX DIFFERENCES IN RELATIONS OF DIABETES BIOMARKERS WITH PHYSICAL FUNCTIONING BEFORE DIABETES ONSET

**DOI:** 10.1093/geroni/igad104.1490

**Published:** 2023-12-21

**Authors:** Tasneem Khambaty, Ashley Splain, Leslie I Katzel, Michele K Evans, Alan B Zonderman, Shari R Waldestein

**Affiliations:** University of Maryland, Baltimore County, Baltimore, Maryland, United States; University of Maryland, Baltimore County, Baltimore, Maryland, United States; Baltimore VA Medical Center, Baltimore, Maryland, United States; National Institute on Aging, National Institutes of Health, Baltimore; National Institute on Aging, National Institutes of Health, Baltimore; University of Maryland, Baltimore County, Baltimore, Maryland, United States

## Abstract

It remains unclear if declines in physical functioning linked to Type 2 diabetes (T2DM) occur even before T2DM onset. We examined the longitudinal associations of T2DM biomarkers with upper and lower extremity strength among adults without T2DM at baseline. Participants were 1,572 African American (AA) and White adults (M(SD) age at baseline = 47.3 (9.7) years, 55% female, 53% AA) from the Healthy Aging in Neighborhoods of Diversity across the Life Span (HANDLS) study, assessed on up to 3 time points between 2004-2017. Participants provided blood samples for measurement of fasting glucose and glycosylated hemoglobin (HbA1c), and completed dominant and nondominant handgrip strength assessment, and a chair stands task to assess upper and lower extremity strength, respectively. Linear mixed effects regression models estimating relations of HbA1c on age-related change and adjusting for race, sex, literacy, and poverty status revealed significant four-way interactions of HbA1c, race, sex, and age (B = -.2.59, t(2853) = -2.0 p = 0.04), and to a lesser extent of fasting glucose, race, sex, and age (B = -.07, t(2863) = -1.84 p = 0.06) on change in the chair stands task, but not on handgrip strength (ps > 0.29). Among younger AA and White men, and younger White women, increasing HbA1c was related to worse lower extremity strength. However, among AA women, and older White women, increasing HBA1c was related to better lower extremity strength. Our findings suggest differential relations of worse glucose regulation on physical functioning amongst African American and White men and women.

